# ABCG1 maintains high-grade glioma survival *in vitro* and *in vivo*

**DOI:** 10.18632/oncotarget.8030

**Published:** 2016-03-10

**Authors:** Yi-Hsien Chen, Patrick J. Cimino, Jingqin Luo, Sonika Dahiya, David H. Gutmann

**Affiliations:** ^1^ Department of Neurology, Washington University School of Medicine, St. Louis, MO, USA; ^2^ Department of Pathology, Washington University School of Medicine, St. Louis, MO, USA; ^3^ Department of Surgery, Washington University School of Medicine, St. Louis, MO, USA

**Keywords:** glioblastoma, ER stress, brain tumor, glioma stem cell, apoptosis

## Abstract

The overall survival for adults with malignant glioma (glioblastoma) remains poor despite advances in radiation and chemotherapy. One of the mechanisms by which cancer cells develop relative resistance to treatment is through de-regulation of endoplasmic reticulum (ER) homeostasis. We have recently shown that ABCG1, an ATP-binding cassette transporter, maintains ER homeostasis and suppresses ER stress-induced apoptosis in low-grade glioma. Herein, we demonstrate that ABCG1 expression is increased in human adult glioblastoma, where it correlates with poor survival in individuals with the mesenchymal subtype. Leveraging a mouse model of mesenchymal glioblastoma (NPcis), shRNA-mediated Abcg1 knockdown (KD) increased CHOP ER stress protein expression and resulted in greater NPcis glioma cell death *in vitro*. Moreover, Abcg1 KD reduced NPcis glioma growth and increased mouse survival *in vivo*. Collectively, these results demonstrate that ABCG1 is critical for malignant glioma cell survival, and might serve as a future therapeutic target for these deadly brain cancers.

## INTRODUCTION

Brain tumors represent the fourth leading cause of cancer-related death in adults, where high-grade glial neoplasms (malignant gliomas) predominate [1]. These malignant gliomas are classified by the World Health Organization (WHO) as grade III (anaplastic astrocytoma, anaplastic oligodendroglioma, and anaplastic mixed oligoastrocytoma) or IV (glioblastoma) astrocytomas, commonly occurring in individuals between the ages of 45 and 75 years [2]. Unfortunately, survival following the diagnosis of a glioblastoma is dismal, with most patients dying within 12–16 months despite aggressive surgical, radiation, and chemotherapy management [1, 3]. Since these cancers are thought to be maintained by cells with stem cell-like properties (glioma stem cells; GSCs), intense focus over the past decade has centered on understanding the critical pathways that govern GSC growth and differentiation [4, 5].

In this regard, a large number of studies have revealed that GSCs are capable of long-term self-renewal, multi-lineage differentiation, and the generation of histologically-similar tumors following implantation into immunocompromised rodent brains [6]. Most often identified by their expression of the prominin-1 cell surface marker, CD133 [6, 7], GSCs can be enriched by antibody-mediated isolation, and shown to possess unique properties such as radioresistance and chemoresistance not shared with normal brain neural stem cells (NSCs) [8–10]. Another of these markers is the ABCG2 protein, which identifies the “side population”, a subset of stem cells hypothesized to be enriched for cancer propagation abilities [9, 11].

Similar to ABCG2, ABCG1 belongs to a large family of ATP-binding cassette proteins involved in cellular transport [12, 13], where it mainly directs lipid transport [14, 15]. In this regard, we recently leveraged a low-grade glioma mouse model of Neurofibromatosis type 1 (NF1)-associated optic glioma to identify and characterize low-grade glioma (LGG) cancer stem cells (LG-GSCs) [16]. Similar to their malignant glioma counterparts, these LG-GSCs were capable of long-term self-renewal, multi-lineage differentiation, and forming low-grade glioma-like lesions following explantation into immunocompetent mice. However, ABCG2 was not highly expressed in these murine LG-GSCs, consistent with an absence of a side population following Hoechst 33342 flow cytometry. Instead, ABCG1 was highly enriched in both murine and human LG-GSCs relative to their non-neoplastic NSC counterparts, where it served to protect these cells from ER stress-mediated cell death (apoptosis).

Based on the controversial use of ABCG2 as a marker of CSCs [11] and the recent identification of ABCG1 as a critical mediator of LG-GSC survival, we sought to determine whether ABCG1 was important for high-grade (glioblastoma) survival. In this report, we demonstrate that *ABCG1* expression negatively correlates with survival in patients with glioblastoma of the mesenchymal subtype. Leveraging a mouse model of mesenchymal glioblastoma (NPcis), characterized by *Nf1* and *Trp53* loss, we show that shRNA-mediated *Abcg1* knockdown increases ER stress-induced apoptosis *in vitro* as well as improves the survival of immunocompetent mice with glioblastoma *in vivo*. Collectively, these results establish ABCG1 as another potential biomarker for high-grade glioma survival relevant to future brain tumor therapeutic targeting.

## RESULTS

### Glioblastoma ABCG1 expression is associated with reduced patient survival

**Figure 1:**
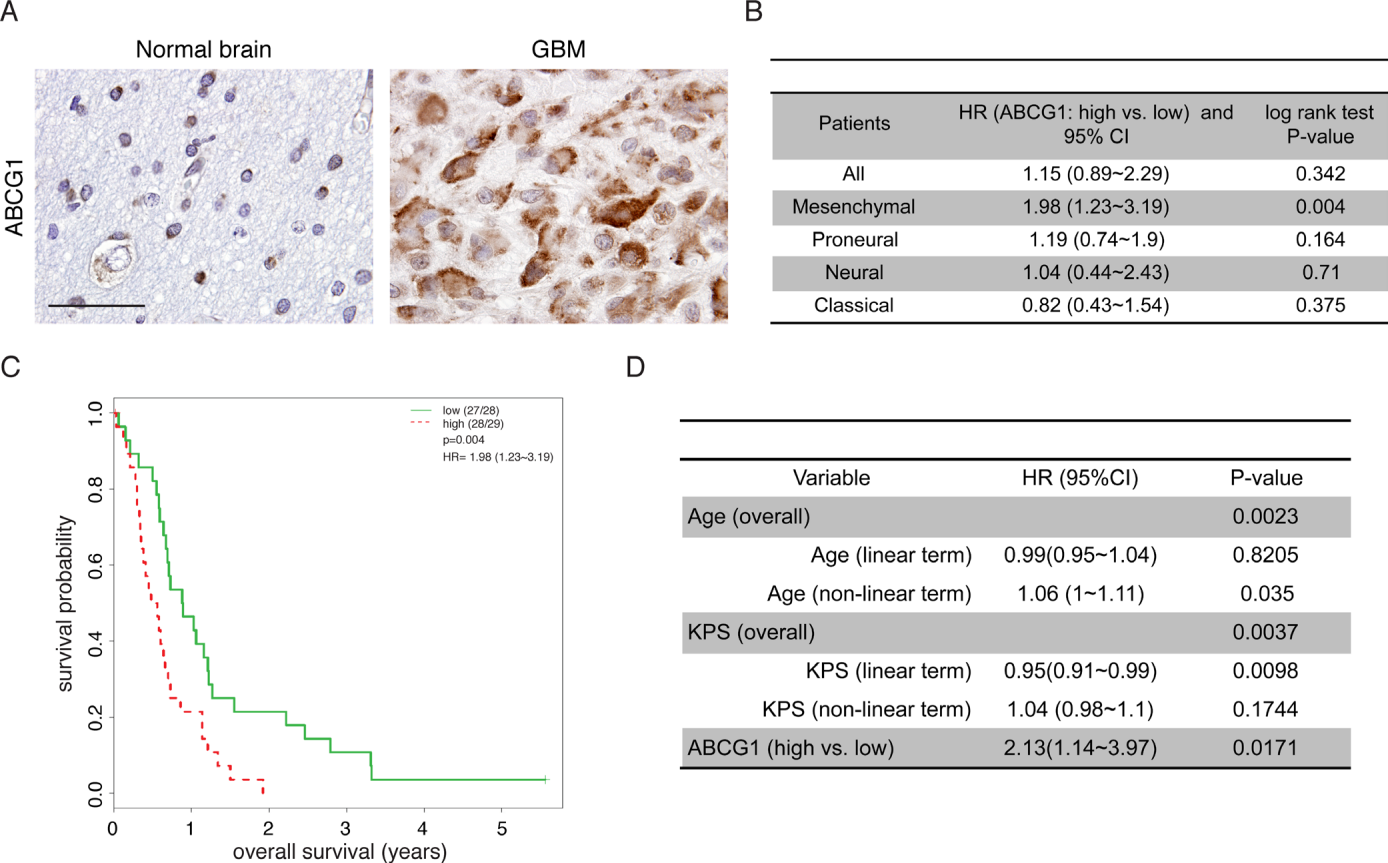
Abcg1 expression is associated with poor overall survival in the mesenchymal glioblastoma molecular subtype (**A**) A representative specimen from one adult with a glioblastoma reveals increased ABCG1 expression relative to one representative age-matched control brain. Scale bar, 50 μm. (**B**) Prognostic effect of ABCG1 (high vs. low) in all patients and within each subtype. (**C**) Kaplan-Meier analysis demonstrates that increased *ABCG1* expression negatively correlated with overall survival in patients with the mesenchymal glioblastoma subtype (*p* = 0.004) in GSE16011. (**D**) In GSE16011, while both age (*P* = 0.0023) and KPS (*P* = 0.0037) are associated with overall survival, multivariate analysis shows that high *ABCG1* expression is a strong predictor of poor patient survival, independent of age and KPS (*P* = 0.017).

We have recently shown that human low-grade (pilocytic astrocytoma) glioma specimens exhibit increased ABCG1 expression [16]. To determine whether ABCG1 expression was similarly increased in human glioblastoma specimens, two sets of experiments were performed. Using the one available GEO dataset containing normal reference tissue (astrocytes; GSE15824), we found that *ABCG1* expression was increased in glioblastoma tumors (697 ± 554, 230913_at probe set; 428 ± 305, 232081_at probe set) relative to astrocytes (11.5 ± 1.1, 230913_at probe set; 13.9 ± 8.1, 232081_at probe set). Similarly, increased ABCG1 immunoreactivity was observed in representative tumor specimens from human patients with glioblastoma (*n* = 16) relative to normal brain controls (*n* = 7) (Figure 1A and S1).

Next, we sought to determine whether *ABCG1* expression was associated with clinical outcome (overall survival) in patients with high-grade glioma (GSE16011), the majority of whom did not receive prior chemotherapy. Using a total of 159 glioblastoma patients dichotomized by the median of *ABCG1* expression, there were no significant survival differences between patients harboring gliomas with low versus high *ABCG1* gene expression (Figure 1B). However, when the analysis was stratified by glioblastoma molecular subtype, high *ABCG1* expression correlated with shorter overall survival only in the mesenchymal subgroup (Figure 1C), a subtype characterized by *NF1* gene mutation [17]. Moreover, using multivariate Cox hazard regression analysis with adjustment for both age and Karnofsky Performance Score (KPS), the negative prognostic value of *ABCG1* expression in mesenchymal GBM patient survival remained significant (*P* = 0.017; Figure 1D). Together, these findings demonstrate increased ABCG1 expression in human malignant gliomas, where it is associated with reduced survival in patients harboring the mesenchymal glioblastoma subtype.

### Abcg1 knockdown decreases glioblastoma cell growth *in vitro*

**Figure 2:**
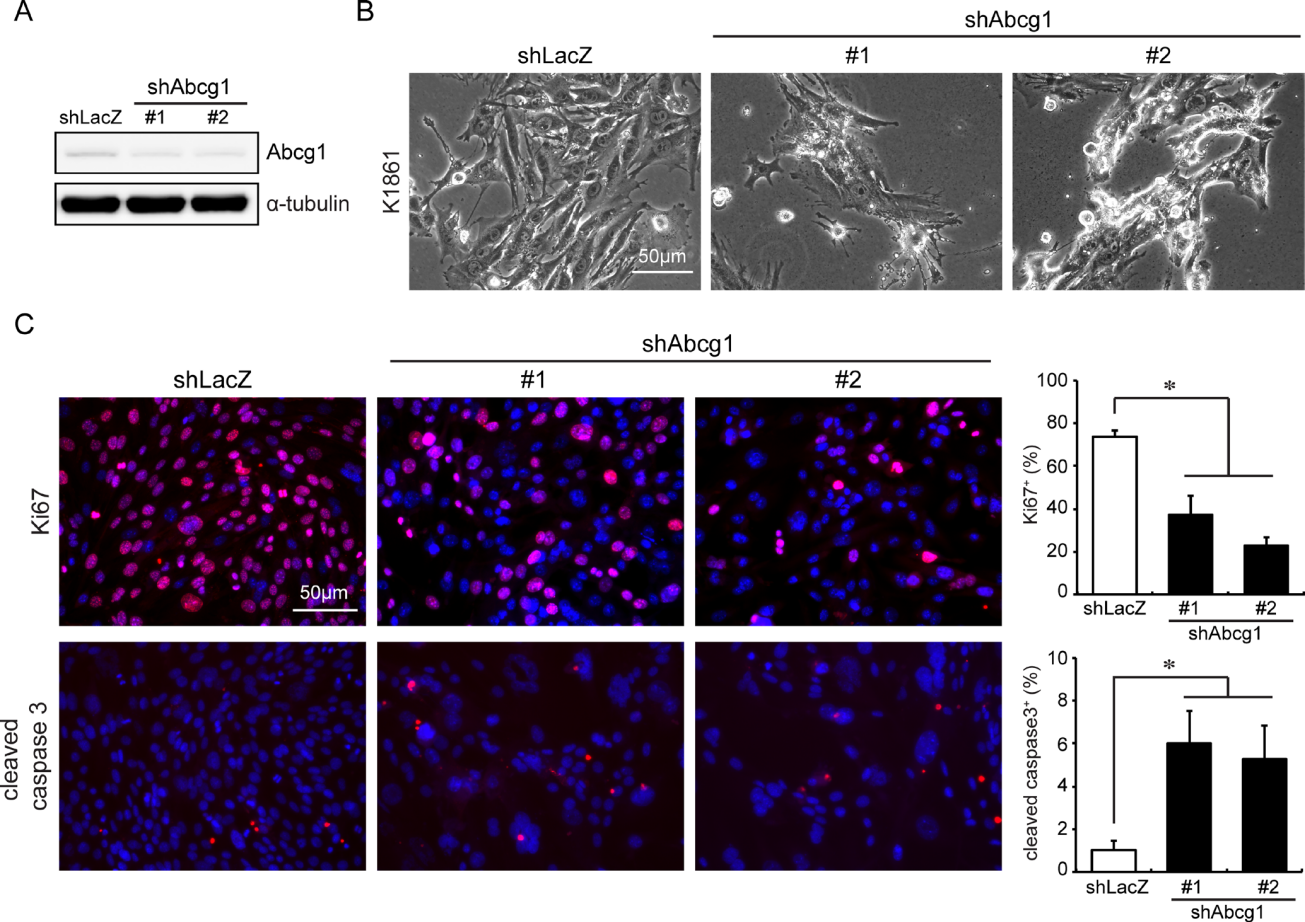
Abcg1 knockdown reduces NPcis glioma cell growth *in vitro* (**A**) shRNA-mediated Abcg1 knockdown in K1861 NPcis glioma cells. (**B**) NPcis cells 6 days post-infection with sh*Abcg1* or control sh*LacZ* virus. Following Abcg1 knockdown, there was (**C**) reduced cell growth (% Ki67^+^ cells) and increased apoptosis (% cleaved caspase 3^+^ cells). Scale bar, 50 μm. Error bars denote mean ± SD. (*) *p* < 0.05.

In the glioblastoma mesenchymal molecular subtype, *NF1* loss is frequently associated with *TP53* mutation [17]. Consistent with this observation, mice harboring germline inactivating mutations in both the *Nf1* and *Trp53* genes residing on the same chromosome (NPcis mice) spontaneously develop high-grade gliomas [18, 19]. Moreover, derivative glioma cells from these NPcis mouse tumors exhibit bi-allelic loss of the *Nf1* and *Trp53* genes in addition to increased PDGFRα expression, molecular features also observed in human mesenchymal glioblastomas [17, 20, 21]. Given the correlation between *ABCG1* expression and overall survival in patients with mesenchymal subtype glioblastoma, we sought to determine whether ABCG1 might be important for NPcis glioma growth using lentivirus-mediated shRNA knockdown in two independently-generated NPcis high-grade glioma cell lines (K1861 and K4622; [22]). First, we confirmed Abcg1 knockdown using two distinct shRNA constructs by Western blotting (Figure 2A; ~50% protein reduction). Following Abcg1 knockdown, K1861 cells exhibited morphological changes (*e.g*., rounding) suggestive of cell death (Figure 2B). Consistent with these cellular changes, reduced cell growth (% Ki67^+^ cells; Figure 2C) and increased apoptosis was observed (% cleaved caspase-3^+^ cells; Figure 2C) relative to shRNA controls. Identical results were also observed using the K4622 high-grade glioma cell line (Figure S2).

### Abcg1 knockdown increases ER stress and glioblastoma cell apoptosis

**Figure 3:**
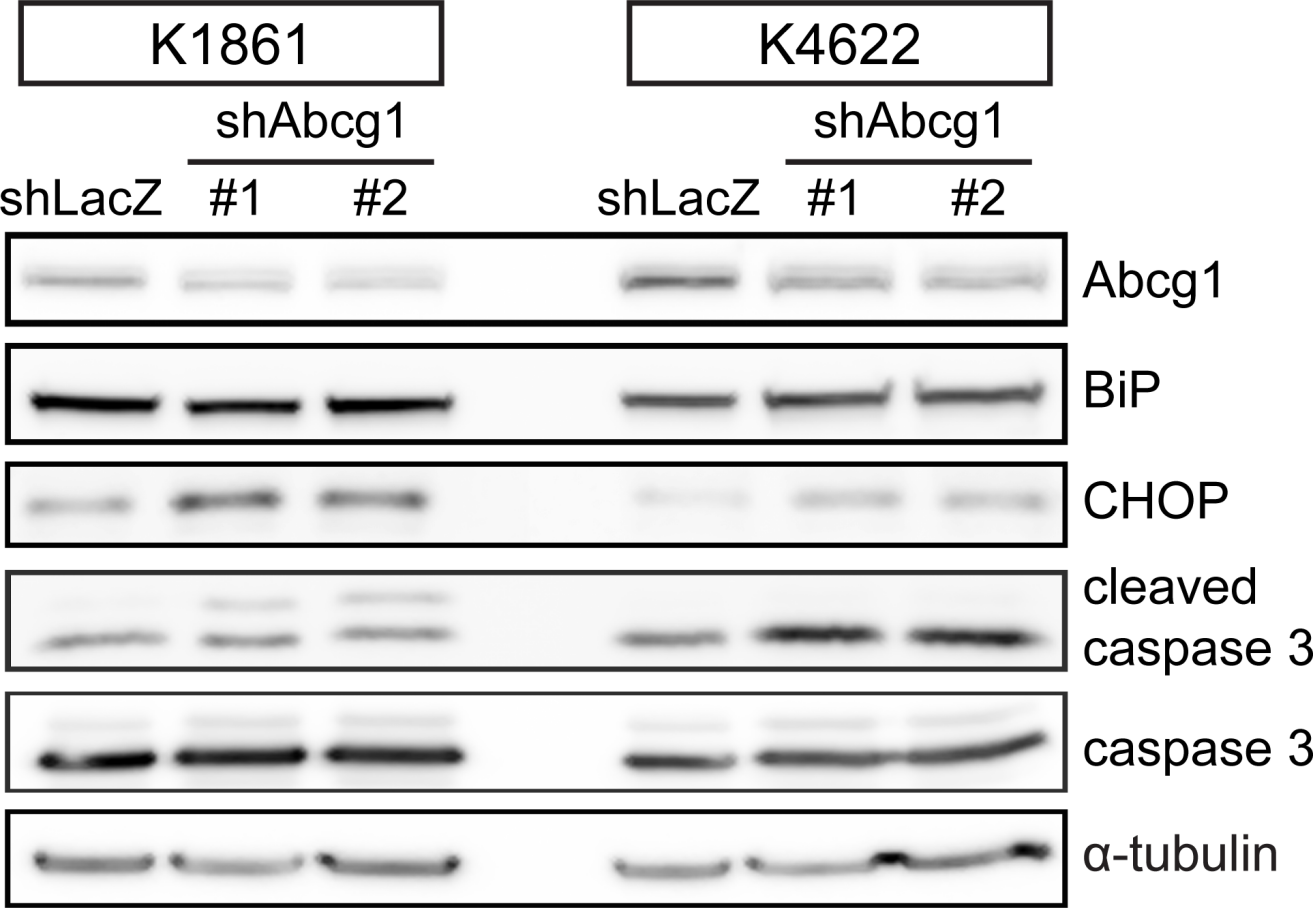
Abcg1 knockdown increases the ER stress response shRNA-mediated Abcg1 knockdown in K1861 and K4622 glioblastoma cells increased CHOP and cleaved caspase-3, but not BiP, expression.

Since Abcg1 confers a survival advantage to LG-GSCs by suppressing ER stress [16], we examined the expression of BiP and CHOP, two markers of ER stress. Following Abcg1 knockdown (45–54% protein reduction) in K1861 and K4622 glioblastoma cells, there was increased CHOP expression (2.3 and 3-fold, respectively) and caspase-3 cleavage (1.5 and 2.2-fold, respectively) relative to controls (Figure 3). However, in contrast to LG-GSCs, there was no change in BiP expression upon Abcg1 reduction.

### Abcg1 knockdown reduces glioma growth and mouse survival *in vivo*

**Figure 4:**
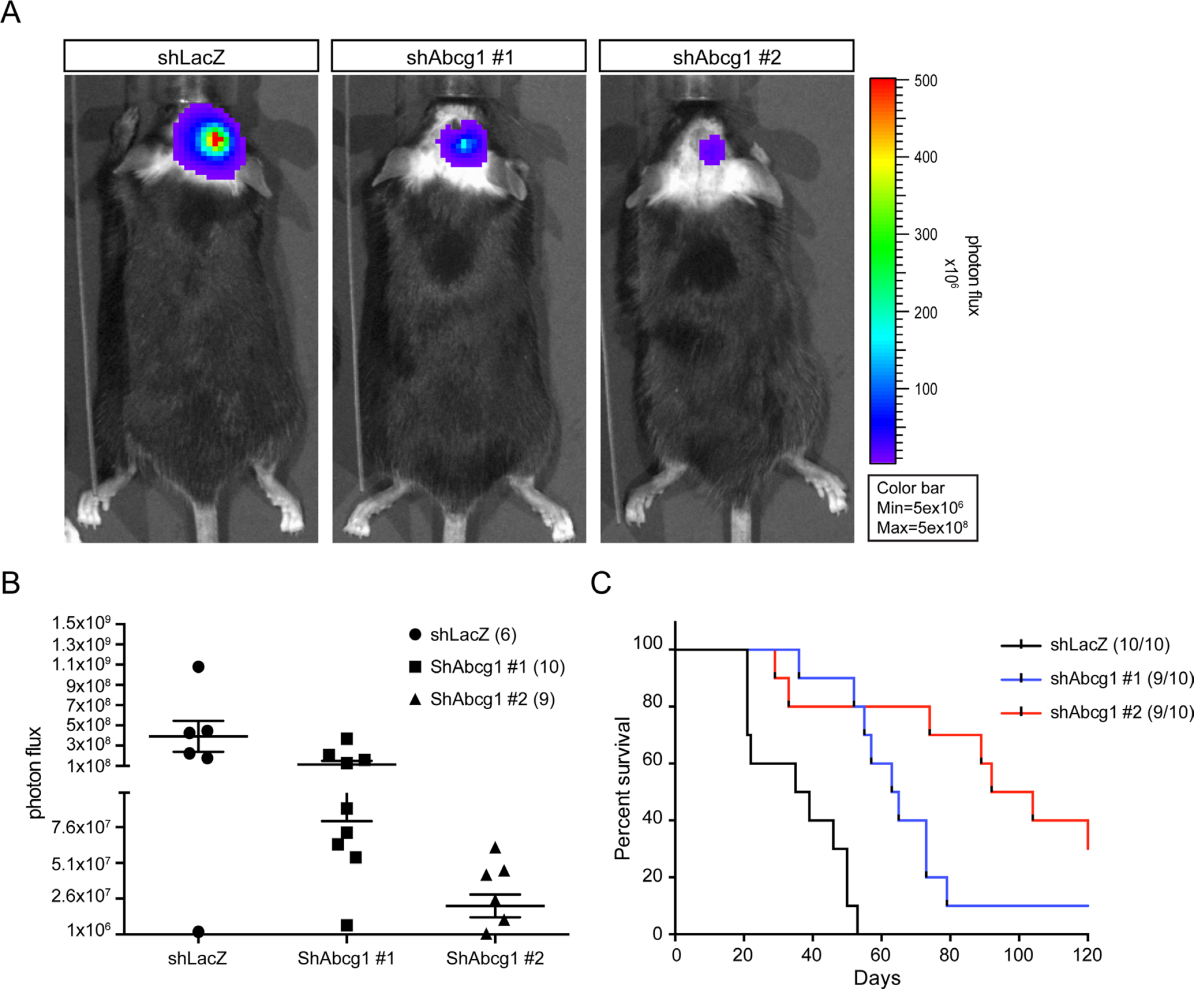
Abcg1 knockdown reduces tumor growth *in vivo* (**A**) Representative bioluminescence images of tumors in engrafted mice from control sh*LacZ* and sh*Abcg1* groups (#1, #2) at 28 day post-transplantation were shown. (**B**) Photon flux of alive mice in all groups at 4 weeks post-transplantation. Relative to sh*LacZ*-K1861 implanted mice, C57BL/6 mice receiving sh*Abcg1*-K1861 tumors had reduced bioluminescence signal (#1, *p* = 0.042; #2, *p* = 0.0098). Error bars denote mean ± SEM. (**C**) Kaplan-Meier survival analysis reveals that mice transplanted with sh*Abcg1* K1861 NPcis cells exhibit longer survival than those injected with control (sh*LacZ*) NPcis cells (log-rank test; *p* < 0.0001).

To determine whether Abcg1 knockdown also decreases glioma growth *in vivo*, 5.0 × 10^4^ K1861 glioblastoma cells were stereotactically injected in the right striatum of 3-week-old C57Bl/6J mice. Since these K1861 cells were transduced with a lentiviral vector expressing green fluorescent protein and firefly luciferase, tumor growth could be monitored by live bioluminescence imaging (BLI). We specifically chose to employ the K1861 glioblastoma for these *in vivo* experiments, based on previous studies demonstrating that K1861-engrafted mice develop large and aggressive intracranial tumors [19, 23]. Whereas all engrafted mice harbored brain tumors, there was reduced tumor growth by BLI in the two K1861 *Abcg1* shRNA groups (*n* = 10 mice/group) relative to control shRNA K1861-implanted mice (*n* = 10 mice) at 28 days post-injection (Figure 4A) and the bioluminescent signal was significantly reduced in both *Abcg1* shRNA groups relative to control shRNA K1861-implanted mice (#1, *p* = 0.042; #2, *p* = 0.0098; Figure 4B). Over the 120 days of observation, mice in the shRNA control group became ill and had to be euthanized (median survival = 37 days). However, in striking contrast, mice receiving *Abcg1*-shRNA K1861 glioblastoma cells had median survivals of 64 (*P* < 0.0001) and 98 (*P* < 0.0001) days, respectively (Figure 4C). While sh*Abcg1*-#2 was slightly more effective at reducing Abcg1 protein expression than sh*Abcg1*-#1 (15% versus 30% of sh*LacZ* levels), which might account for the observed lower BLI signal observed in mice receiving 1861 cells with shAbcg1-#2 versus shAbcg1-#1 (*P* = 0.0226), there was no difference in survival (*P* = 0.1426).

**Figure 5:**
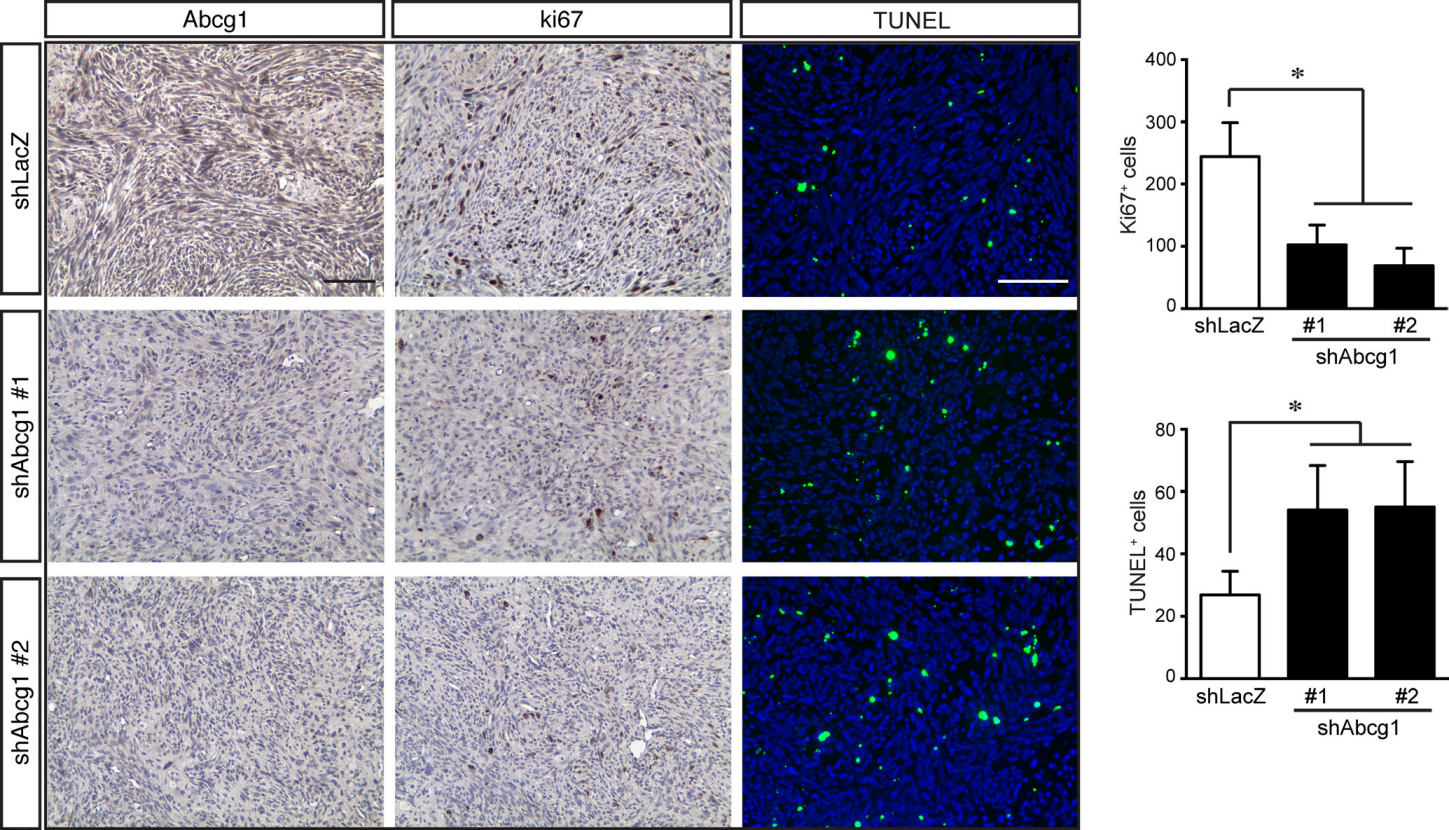
Abcg1 knockdown increases glioblastoma apoptosis *in vivo* Glioma sections from control mice (shLacZ) reveal reduced Abcg1 immunostaining relative to mice harboring tumors with sh*Abcg1* knockdown. Following Abcg1 knockdown, there was decreased glioma growth (% Ki67^+^ cells) and increased apoptosis (% TUNEL^+^ cells). Error bars denote mean ± SD. (*) *p* < 0.05.

To determine whether Abcg1 knockdown resulted in increased glioblastoma apoptosis *in vivo*, Ki67 and TUNEL immunostaining were performed on the tumors following euthanasia. Consistent with the above *in vitro* findings, shAbcg1 K1861 tumors had reduced Abcg1 protein expression and fewer Ki67^+^ cells relative to controls (Figure 5). In addition, shAbcg1 K1861 tumors had increased cell death (TUNEL^+^ cells). Collectively, these findings establish Abcg1 as a mediator of ER stress-mediated apoptosis and survival in high-grade glioma.

## DISCUSSION

Adaption to ER stress represents one protective mechanism for sustaining tumor cell growth and survival in the setting of hypoxia, low glucose levels, or chemotherapeutic drug exposure [24, 25]. While little is known about ABCG1 in cancer, we demonstrate for the first time that ABCG1 is expressed in both murine and human glioblastoma, where its expression correlates with overall patient survival. Interestingly, *ABCG1* expression was not associated with survival in patients with all molecular subtypes of glioblastoma; however, there was a strong correlation between poor patient survival and high *ABCG1* RNA expression in tumors classified in the mesenchymal GBM subtype. In this subtype, one of the signature genetic changes is mutation of the *NF1* tumor suppressor gene [17], which is intriguing, given the prior identification of ABCG1 as a uniquely upregulated transcript in GSCs originating from a mouse model of low-grade glioma harboring biallelic *Nf1* gene inactivation [16, 26]. Importantly, increased Abcg1 expression does not result from *Nf1* loss directly, but is rather a consequence of GSC development. In this regard, only optic glioma stem cells, but not *Nf1*-deficient NSCs, exhibit increased Abcg1 expression [16]. While it is currently not known how ABCG1 expression is regulated, a recent study conducted in CHO cells implicated E3-ubiquitin ligases (HUWE1 and NEDD4-1) in the post-translational regulation of ABCG1 protein stability and cholesterol export [27]. Formal demonstration of an ubiquitin ligase pathway that coordinately regulates neurofibromin and ABCG1 expression in glioma warrants further investigation [28, 29].

Similar to our findings in LG-GSCs, reduced Abcg1 protein expression using two different shRNA targeting constructs in two independently-generated NPcis glioblastoma lines (K1861 and K4622) increased the expression of CHOP, a final effector of the ER stress response, and resulted in higher levels of tumor cell death by apoptosis. However, in contrast to optic glioma stem cells, some molecular elements of the ER stress pathway were not conserved in glioblastoma cells. In this regard, we did not observe any changes in BiP expression, PDI expression, or PERK phosphorylation (data not shown), suggesting that other ABCG1-dependent mechanisms for inducing ER stress may be operative. Further studies focused on additional ER stress pathways in glioblastoma are currently being explored.

The fact that attenuated ABCG1 expression significantly prolongs mouse survival following glioblastoma implantation supports a critical role for this protein in tumor maintenance. This *in vivo* effect was demonstrated both in terms of tumor growth (bioluminescence imaging and tumor proliferation) and apoptosis (TUNEL), reminiscent of reported effects of ABCG2 in high-grade brain tumors. In these studies, ABCG2 has been implicated in reduced brain penetration of enhancer of zeste homolog 2 (EZH2) inhibitors [30] as well as the anti-tumoral responses to PARP inhibitors (ABT-888) and temozolomide [31]. Moreover, ABCG2 downregulation inhibits glioma stem cell migration and invasion [32, 33], where its expression partly dictates the behavior of the side population of high-grade GSCs [9, 34]. These exciting observations prompted recent discovery efforts to identify potential modulators of ABCG2 [35]. As the etiologic mechanisms responsible for ABCG1-mediated suppression of ER stress and glioma survival become elucidated [36–41], future therapeutic strategies designed to blocking these adaptations may improve treatment efficacy for this deadly brain malignancy.

## MATERIALS AND METHODS

### Cell lines and mice

Mouse malignant glioma NPcis cell lines, K1861 and K4622, were derived from C57BL/6J *Trp53*^+/−^/*Nf1*^+/−^ mice (Dr. Karlyne Reilly, National Cancer Institute) [22]. These lines were maintained at 37°C in 5% CO_2_ in Dulbecco's modified Eagle medium containing 10% fetal bovine serum. All mouse procedures were performed in accordance with an approved Animal Studies protocol at Washington University.

### Lentivirus infections

Prior to the lentiviral sh*Abcg1* infection, NPcis cells were transduced with lentiviral FUW-GL vector (Dr. Joshua Rubin) for bioluminescence imaging. *Abcg1* shRNA or control shLacZ-containing pLKO.1 plasmid was co-transfected with pMDLg/pRRE, pRSV-REV and pCMV-VSV-G into HEK293T cells using the FuGENE HD transfection reagent (Roche). NPcis cells were dissociated into single cells by trypsin and transduced with viral supernatants from HEK293T cells with polybrene (8 μg/mL) for 48 hours. The knockdown efficiency of the *Abcg1* shRNA constructs was evaluated by Western blotting. Lentiviral constructs used are listed in Table S1.

### Western blotting

Western blotting was performed as previously reported [42] using the primary antibodies listed in Table S2.

### Intracranial injections

Injections were performed as previously described [43]. 3 to 5-week-old male wild-type C57BL/6J purchased from Taconic were used. NPcis cells were prepared in a 2.5 × 10^4^ cell/μL PBS solution and 2 μL (5 × 10^4^) cells were sterilely implanted intracranially into the right side striatum using a stereotactic device (coordinates = posterior 3 mm from the bregma, lateral 1.5 mm (right), and depth 2.5 from dura mater). Mice were monitored and euthanized after neurological symptoms developed.

### *In vitro* cell proliferation and death

NPcis cells were trypsinized and plated onto 50 μg/mL poly-D-lysine-coated and 10 μg/mL fibronectin-coated 24-well plates in defined culture medium. After 24 hours, cells were fixed in 4% paraformaldehyde. Proliferating and apoptotic cells were detected using antibodies to Ki67 and cleaved caspase-3, respectively. The percent of Ki67^+^ or cleaved caspase-3^+^ cells were determined as a percent of the total cell number (DAPI^+^ cells).

### Human specimens and immunostaining

The use of human subject materials was approved by the institutional review board of the Washington University School of Medicine. Sixteen intracranial glioblastomas (WHO grade IV) and seven surgically obtained brain control cases were identified. Corresponding formalin-fixed paraffin-embedded blocks from the pathology archives were used for immunohistochemistry. Paraffin sections were processed [44] prior to staining with appropriate antibodies (Table S2). The TUNEL labeling was performed using a fluorescence-based *in situ* cell death detection kit (Roche Diagnostics). The numbers of Ki67^+^ and TUNEL-positive cells were counted within the tumors per surface area (0.1 mm^2^).

### Bioluminescence imaging

Animals were given 150 μg/mL D-luciferin (Gold Biotech) in PBS through intraperitoneal injection and imaged with a charge-coupled device (CCD) camera-based bioluminescence imaging system (IVIS50; Perkin-Elmer, Hopkinton, MA) with exposure time 10 secs - 1 minute, binning 8, field of view 12, f/stop 1, open filter. Signal was displayed as photons/sec/cm^2^/sr [45].

### Survival outcome analyses

The survival outcome analyses were performed on the GSE16011 dataset using the R package “RMS”. Each GBM sample (159 out of 276 total samples) was assigned to a TCGA subtype (Classical, Mesenchymal, Neural, or Proneural) based on the 10-nearest neighbors algorithm as previously described [46]. Kaplan-Meier survival plots were generated, along with log rank tests, to compare survival differences between the low/high-expression groups. Multivariate Cox models were fitted to gene expression (binary format, as dichotomized by the median) with incorporation of classic clinicopathological parameters (*e.g*., TCGA molecular subtype, age and KPS) to assess the independent prognostic ability of the candidate gene by the adjusted hazard ratio and associated 95% confidence interval (CI). Age and KPS were incorporated in the format of restricted cubic spline basis function with three knots in order to relax the linearity assumption.

### Statistical analysis

Each experiment was performed with samples from at least three independent groups. All data were processed and graphed in Prism GraphPad 6.0 with descriptive statistics calculated. The difference between experimental groups was assessed by one-way ANOVA. Survival curves were generated by the Kaplan-Meier method, and the Log-rank (Mantel-Cox) test was used to compare between/among treatments. All tests were two-sided with a significance level of 5% unless otherwise noted.

## SUPPLEMENTARY MATERIALS FIGURES AND TABLES


