# Supplementary material for: ABCG1 maintains high-grade glioma survival in vitro and in vivo
Source: Oncotarget. 2016 Mar 10;7(17):23416–24. doi: 10.18632/oncotarget.8030 (PMC5029636; doi:10.18632/oncotarget.8030)
Supplement: Supplementary file 1 [file oncotarget-07-23416-s001.pdf]

ABCG1 maintains high-grade glioma survival *in vitro* and *in vivo*

Supplementary Materials

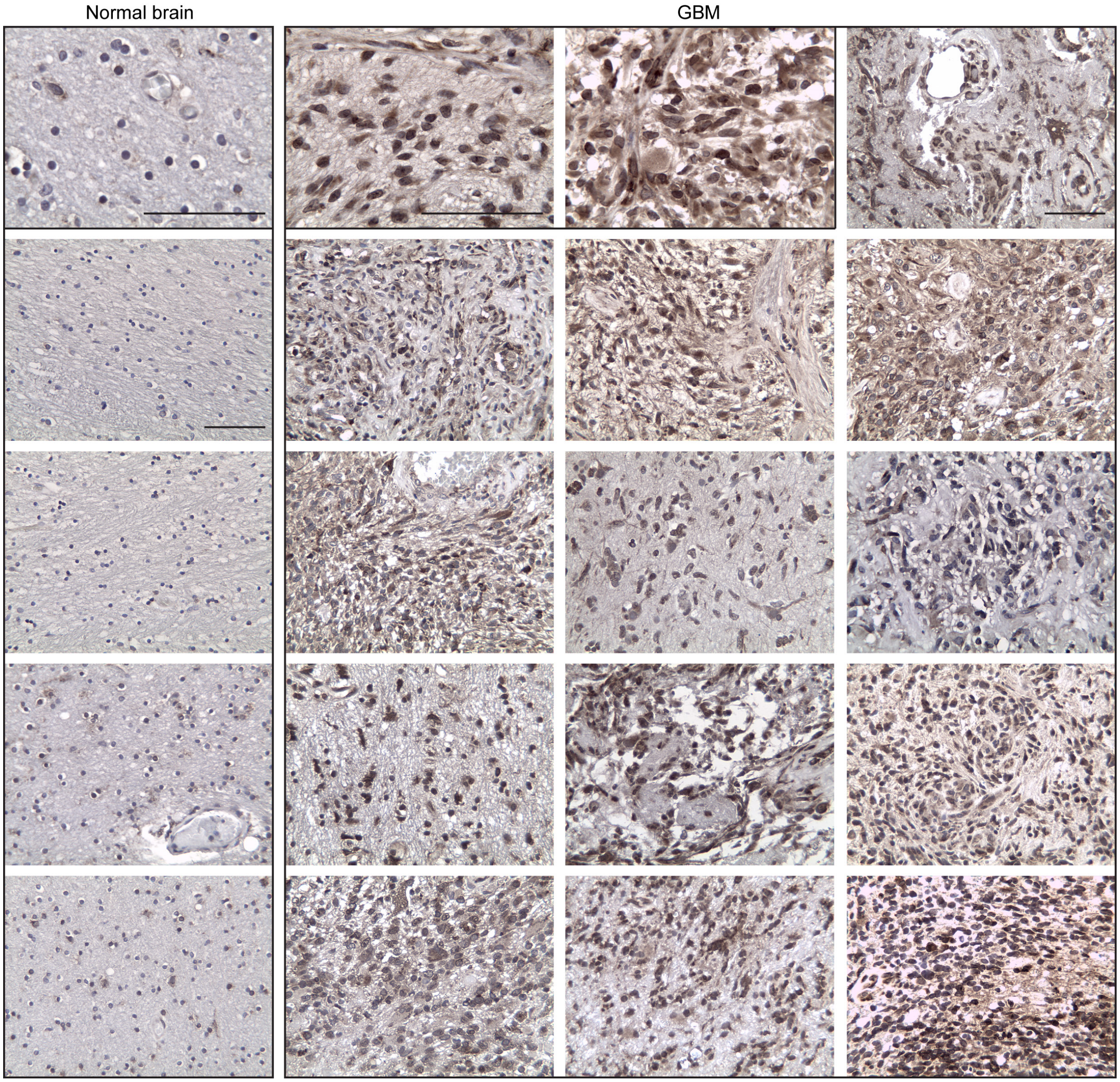

Supplementary Figure S1: Human GBM specimens exhibit strong ABCG1 immunostaining. Representative images from thirteen GBM patients show increased ABCG1 staining. Normal human brain (*n* = 4) was used as a reference control. 200× magnification. Representative normal brain (first specimen at top) and GBM tumors (first two specimens in the top row) images are shown at 400× magnification, demonstrating negative and positive cell staining, respectively. Scale bar: 200 µm.

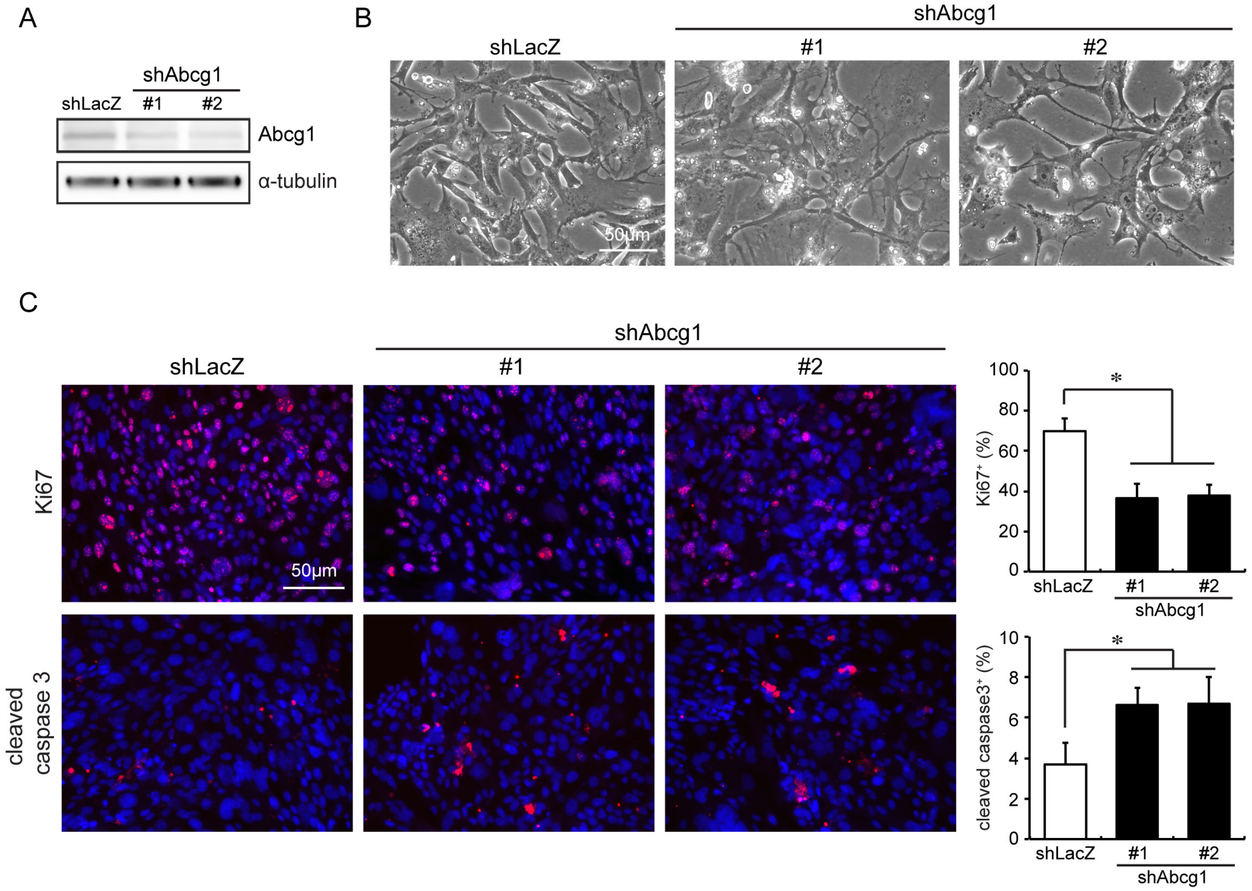

Supplementary Figure S2: Abcg1 knockdown reduced NPcis glioma cell growth in vitro. (**A**) shRNA knockdown reduced Abcg1 expression (65% protein reduction) in K4622 glioma cells. (**B**) K4622 cells were imaged under phase microscopy 6 days post-infection with shAbcg1 (#1 or #2) or control shLacZ virus. (**C**) Abcg1 knockdown decreased K4622 cell growth (% Ki67^{+} cells) and survival (apoptosis; % cleaved caspase 3^{+} cells). Scale bars, 50 μm. Error bars denote mean ± SD. (*) $p < 0.05$.

Supplementary Table S1: Plasmids

Construct	Source
shAbcg1 NM_009593.1-1161s1c1	The Genome Institute at Washington University
shAbcg1 NM_009593.1-1160s1c1	The Genome Institute at Washington University
shLacZ	The Genome Institute at Washington University
FUW-GL	Dr. Joshua Rubin, Washington University

Supplementary Table S2: Antibodies

Antibody	Host	Source	Dilution
Abcg1 (WB, IHC)	Rabbit	GeneTex<br>Abcam	WB 1:2000<br>IHC 1:50
BiP (WB)	Rabbit	Cell Signaling	1:1000
Caspase 3 (WB)	Rabbit	Cell Signaling	1:1000
Cleaved caspase 3 (WB, ICC)	Rabbit	Cell Signaling	WB 1:1000<br>ICC 1:500
CHOP (WB)	Mouse	Cell Signaling	1:1000
Ki67 (IHC, ICC)	Mouse	BD Pharmingen	1:500
α-tubulin (WB)	Mouse	Sigma	1:20,000

WB: Western Blot, IHC: Immunohistochemistry.
